# Novel vaccines for allergen-specific immunotherapy

**DOI:** 10.1097/ACI.0000000000000706

**Published:** 2020-12-02

**Authors:** Oluwatoyin Akinfenwa, Azahara Rodríguez-Domínguez, Susanne Vrtala, Rudolf Valenta, Raffaela Campana

**Affiliations:** a Division of Immunopathology, Department of Pathophysiology and Allergy Research, Center for Pathophysiology, Infectiology and Immunology, Medical University of Vienna, Vienna, Austria; b NRC Institute of Immunology FMBA of Russia; c Laboratory for Immunopathology, Department of Clinical Immunology and Allergy, Sechenov First Moscow State Medical University, Moscow, Russia; d Karl Landsteiner University of Health Sciences, Krems, Austria

**Keywords:** allergen, allergen-specific immunotherapy, allergy, allergy prophylaxis, allergy vaccines, modified allergen molecules, molecular allergy diagnosis

## Abstract

Allergen-specific immunotherapy (AIT) is a highly economic, effective and disease-modifying form of allergy treatment but requires accurate prescription and monitoring. New molecular approaches are currently under development to improve AIT by reducing treatment-related side effects, cumbersome protocols and patients’ compliance. We review the current advances regarding refined diagnosis for prescription and monitoring of AIT and the development of novel molecular vaccines for AIT. Finally, we discuss prophylactic application of AIT.

**Recent findings:**

There is evidence that molecular allergy diagnosis not only assists in the prescription and monitoring of AIT but also allows a refined selection of patients to increase the likelihood of treatment success. New data regarding the effects of AIT treatment with traditional allergen extracts by alternative routes have become available. Experimental approaches for AIT, such as virus-like particles and cell-based treatments have been described. New results from clinical trials performed with recombinant hypoallergens and passive immunization with allergen-specific antibodies highlight the importance of allergen-specific IgG antibodies for the effect of AIT and indicate opportunities for preventive allergen-specific vaccination.

**Summary:**

Molecular allergy diagnosis is useful for the prescription and monitoring of AIT and may improve the success of AIT. Results with molecular allergy vaccines and by passive immunization with allergen-specific IgG antibodies indicate the importance of allergen-specific IgG capable of blocking allergen recognition by IgE and IgE-mediated allergic inflammation as important mechanism for the success of AIT. New molecular vaccines may pave the road towards prophylactic allergen-specific vaccination.

## INTRODUCTION

The burden of allergy is increasing globally and there is need for new therapies that improve the quality of life of allergic patients, reduce economic costs and are suitable for a precision approach [1–3,4^▪^,^5^]. In 1911, Noon [6] was the first to show the benefit of AIT by administering the causative allergen to grass pollen allergic patients to treat the disease. AIT is associated with the induction of protective allergen-specific blocking IgG antibodies and cellular immune mechanisms [7,8^▪▪^]. AIT is the only treatment that instructs the immune system of the patients for protection by therapeutic vaccination and can prevent the progression of the severity of allergic disease [9]. However, the use of crude allergens bears many inconveniences just as induction of side effects, lack of standardization resulting in poor immunogenicity, dose efficacy problems and limited efficacy. Moreover, low patients’ compliance because of cumbersome treatment protocols in particular for sublingual immunotherapy (SLIT) [10,11^▪^,^12^–^14^] is also considered key challenges for traditional AIT.

Advancements in molecular allergen characterization by DNA technology led to the development of new forms of AIT based on recombinant purified proteins, hypoallergenic derivatives and peptides [11^▪^,^15^,^16^]. Moreover, biologics, such as monoclonal IgE antibodies and novel adjuvants have been considered for improving AIT in the last years [17].

**FIGURE 1:**
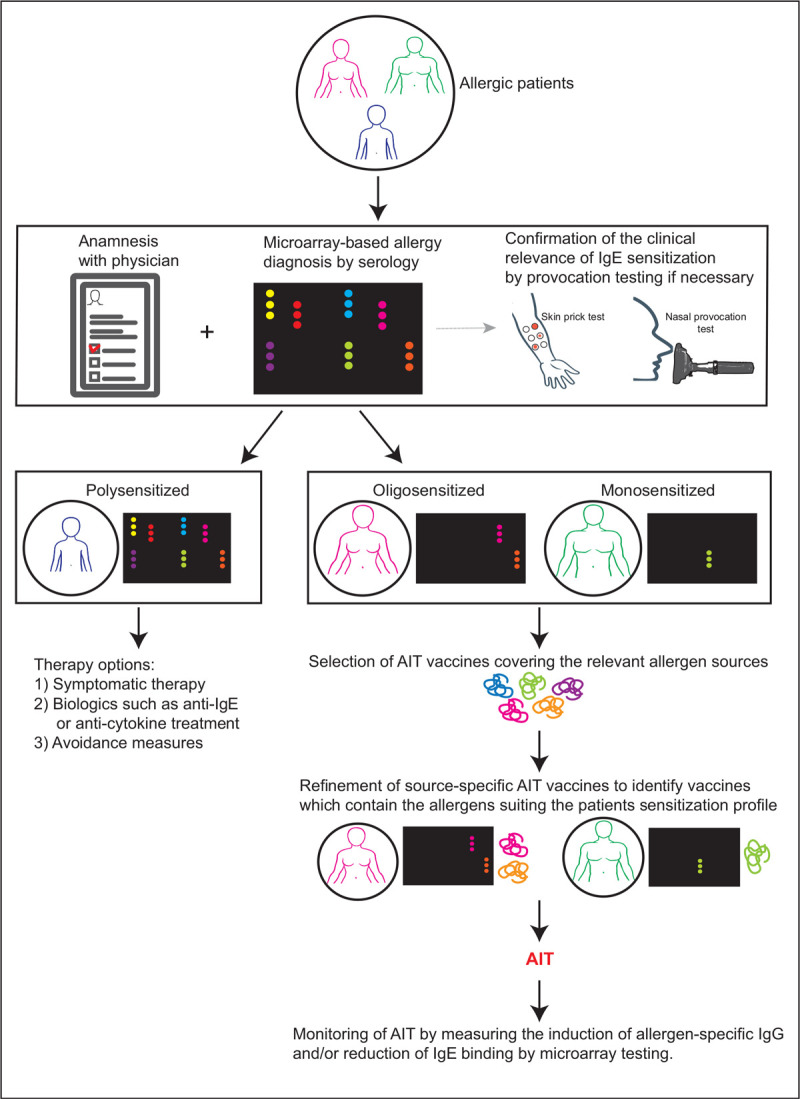
Precision medicine approach to allergy treatment by molecular allergy diagnosis. Molecular diagnosis identifies oligo-sensitized and mono-sensitized patients for AIT, guides prescription of AIT, allows refined selection of vaccines and monitoring of treatment effects. AIT, allergen-specific immunotherapy.

**FIGURE 2:**
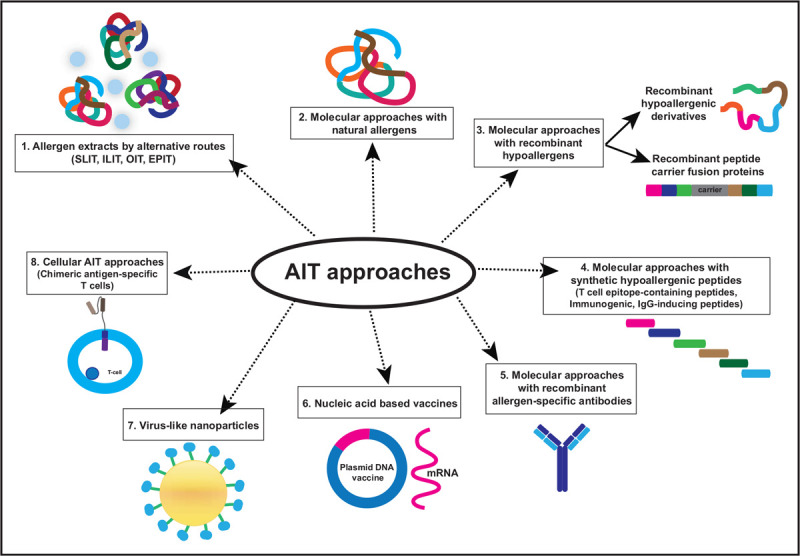
Overview of allergen-specific immunotherapy approaches including different application routes with allergen-extract-based vaccines, molecular AIT approaches, nucleic acid-like, virus-like nanoparticle-based approaches and cellular forms of treatment. AIT, allergen-specific immunotherapy.

This review highlights advances in the field of AIT placing an emphasis on molecular diagnosis for improving prescription and monitoring of AIT (Fig. 1) as well as recent approaches for AIT (Fig. 2 and Table 1). Furthermore, possible approaches for prophylactic vaccination against allergy are considered. 

**Table 1:** Overview of some current allergen-specific immunotherapy-related publications

Approach	Description of approach (references)	Major results
Allergen extract-based AIT by alternative routes	Route: SLIT Description: DBPC Phase III trial. More than 1400 HDM allergic patients received a tablet containing a mixture of *Dermatophagoides pterronyssinus* (Dp) and *Dermatophagoidesfarinae* (Df) extract for 1 year Reference: Pascal *et al.*[33^▪▪^]	Improvement of total combined score in tablet versus placebo group Low increase in allergen-specific IgGs in active group Strong increase in allergen-specific IgE Dp-specific baseline levels (14.81 kUA/l) to median levels of DP-specific IgE (44.01 kUA/l) Treatment-related side effects in more than 50% of active patients
	Route: ILIT Description: DBPC randomized trial with 30 patients allergic to birch pollen or timothy grass Patients received three preseasonal IL allergen injections with 1000 SQ-U of birch or grass pollen extracts in 4 weeks intervals. Active group received a booster dose (1000 SQ-U) 1 year later before the pollen season Reference: Konradsen *et al.*[35^▪▪^]	Nasal symptom scores after NPT decreased 30% in actively treated group versus only 12.5% in placebo group compered with before treatment Strong reduction in MSs in active treated group after the first pollen season (23.1%) and after the booster injection (40%) compared with before treatment Reduction in SSs for active (46.7%) and placebo (18.8%)-treated group Induction of allergen-specific IgG and IgG4, however, the increases were modest compared with SCIT-induced levels
	Route: ILIT Description: 3-year follow up DBPC trial in 36 grass pollen rhinoconjunctivitis patients Study groups: 4 ILIT injections: 3 ILIT + 1 ILIT booster 1 year later, 3 ILIT injections: 3 ILIT + 1 placebo booster 1 year later, Placebo:3 placebo + 1placebo booster 1 year later Active Treatment: 1000 SQ-U *Phleum Pratense*, Alutard (ALK-Abelló). Placebo treatment: isotonic saline Reference: Skaarup *et al.*[36^▪▪^]	cSMS was reduced in 48.5% in the entire 3-year follow-up study period. The booster injection had no additional effect Induction of IgE to grass, IgG4 to grass and Phl p 5 but not IgG4 to Phl p 1
	Route: EPIT Description: DBPC randomized phase III trial accessing the efficacy and safety of peanut patch in 356 children (4–11 years) for 1 year. Active treatment: 250 μg peanut patch Reference: DunnGalvin *et al.*[37^▪▪^]; Fleischer *et al.*[38^▪▪^]	Improvement in food allergy quality of life (FAQL) in active treated patients Adverse reactions: active = 95.4%, placebo = 89%.
	Route: EPIT Description: DBPC randomized trial accessing the clinical efficacy, safety and immunologic effects of EPIT for peanut allergy Patients age: 4–25 years. Active Treatment: Viaskin Peanut 100 μg (*n* = 24) or 250 μg (*n* = 25) Primary outcome: achievement of 10-fold or greater increase in peanut consumption at week 52 compared with baseline Reference: Jones *et al.*[41]	Increase in eliciting dose to peanut protein consumed by treated patients, especially among younger children (age 4–11 years) Increase in peanut-specific IgG4 and trend towards reduced basophil activation and peanut-specific Th2 cytokines
	Route: EPIT Description: evaluation of serological changes in children treated with a 250 μg peanut patch (*n* = 25) or with a placebo patch (*n* = 26) Reference: Koppelman *et al.*[42^▪▪^]	EPIT application induced Increase in peanut-specific IgG4 to Ara h 1, Ara h 2, Ara h 3 and Ara h 6.
	Route: OIT Description: DBPC randomized phase II trial with 120 peanut allergic, age: 7–55 years Groups: peanut 0 (*n* = 60), peanut 300 (*n* = 35), placebo (*n* = 25) Primary outcome: achievement of 4000 mg peanut consumption at weeks 104/117 versus baseline Reference: Chinthrajah *et al.*[44]	Peanut OIT could desensitize individuals with peanut allergy to 4000 mg peanut consumption Common adverse events were mild gastrointestinal symptoms and skin disorders but both decreased over time
	Route: OIT Description: evaluation of blocking antibodies and IgE antibodies induced following peanut allergy treatment. Patients: 22 peanut allergic children, age: 7–17 years Reference: Santos *et al.*[45^▪▪^]	Peanut OIT induced blocking antibodies, which suppressed mast cell activation Peanut IgG4/IgE ratio was increased with treatment Functionality of peanut specific IgE was not altered by the treatment
	Route: OIT Description: DBPC randomized phase III trial in 496 peanut allergic patients (age: 4–17 years) Groups: AR101 (*n* = 372), placebo (*n* = 124). Primary outcome: patients who could consume a single dose of up to 600 mg or more as a challenge dose Reference: PALISADE Group of Clinical Investigators *et al.*[46]	Treatment resulted in higher consumption dose of peanut without dose-limiting symptoms or lower symptom with food challenge Common adverse affected the gastrointestinal and respiratory tracts, skin and the immune system
	Route: OIT Description: evaluation of safety and efficacy of oral immunotherapy for peanut allergy Reference: Chu *et al.*[47]	OIT increases risk of anaphylaxis and other serious adverse events compared with avoidance or placebo
Molecular approaches with natural allergens	Route: SCIT Description: DBPC randomized trial of AIT with the major allergen Alt a 1 (*Alternaria alternata*) Patient: 111 peanut allergic patients, age: 12–65 years Groups: low dose: 0.2 μg Alt 1 (*n* = 37), high dose: 0.375 μg Alt 1 (*n* = 45), placebo (*n* = 29) Reference: Tabar *et al.*[52^▪▪^]	Significant reduction in combined symptom and medication score in the group treated with the highest dose Increased IgG4/IgE ration in both treatment groups Reduced cutaneous reaction to Alt 1 in both treatment groups compared with placebo
	Route: SCIT Description: investigation of immunologic mechanisms in patients who participated in a DBPC phase III immunotherapy trial with *Lolium perene* peptide (LPP) Groups: LPP (*n* = 21), placebo (*n* = 11) Grass-pollen induced basophil, T-cell and B-cell responses were evaluated before treatment, at the end of treatment and after the pollen season Reference: Sharif *et al.*[53^▪▪^]	CSMS were lower during the peak season and throughout the season in LPP-treated group Decrease in CD63+ and CD203c activation in LPP group LPP immunotherapy-induced FoxP3+, follicular Treg cells, and IL-10 reg B cells Induction of regulatory B cells was associated with allergen-neutralizing IgG4
	Route: SCIT Description: evaluation of safety, efficacy and immune mechanisms of short-course treatment with adjuvant-free *Lolium perene* peptides (LPP) Study designed: 61 grass pollen-allergic patients received two s.c. injections of LPP once weekly for 6 weeks. Safety was accessed by CPT, specific-IgE, IgG4 and blocking antibodies before, during and after treatment Reference: Mösges *et al.*[54]	LPP immunotherapy appeared safe with reduced CPT reactivity LPP immunotherapy induced blocking antibodies as early at 4 weeks after treatment
	Route: SCIT Description: DBPC randomized trial with peptide hydrolysates from *Lolium perene* in 554 adults suffering from grass pollen rhinoconjunctivitis Study designed: eight s.c. injections administered in increasing doses of 170 μg LPP in four visits over 3 weeks. Primary outcomes measured: CSMS over the peak pollen, CPT, QOL Reference: Mösges *et al.*[55]	LPP immunotherapy reduced CSMS, CPT and improved QOL
	Route: SCIT Description: phase III randomized multicenter trial of gp-ASIT (*n* = 650) Study: patients were administered 3 weeks before grass pollen season Primary objective: 0.3 absolute reduction in CSMS in treated group compare with placebo group during the peak of the grass pollen season Reference: ASIT biotech gp-ASIT Phase III in grass pollen (https://www.businesswire.com/news/home/20191124005103/en/ASIT-biotech-gp-ASIT%E2%84%A2-Phase-III-Trial-Grass)	ASIT immunotherapy showed only 0.15--0.18 reduction in CSMS during the peak and entire grass pollen season not reaching primary end point
Recombinant hypoallergenic molecules	Route: SCIT Description: DBPC multicenter randomized AIT trial with BM32, a grass pollen allergy vaccine consisting of nonallergenic peptides from Phl p 1, Phl p 2, Phl p 5 and Phl p 6 fused with the hepatitis B preS protein Study design: Grass pollen allergic patients were randomized and received three preseasonal injections of BM32 or placebo + booster injection in autumn in year 1 and 3 preseasonal injections in the second year of the study Evaluation of clinical efficacy by using CSMS, visual analog scales rhinoconjunctivitis QOLQ and asthma symptom scores (Niederberger *et al.*[64]) Related studies Study on the effects of BM32 vaccination and natural allergen exposure on allergen-specific antibody, T cell and cytokine responses (Eckl-Dorna *et al.*[67^▪▪^]) Study on the magnitude and specificity of allergen-specific IgG responses in patients treated with BM32 versus allergen-extract SCIT (Rauber *et al.*[66^▪▪^]) Evaluation of hepatitis B-specific antibody and T cell responses of BM32 treated patients (Cornelius *et al.*[63]) Quantification, epitope mapping and investigation of HBV genotype cross-reactivity of preS-specific antibodies in subjects treated with BM32 (Tulaeva *et al.*[65^▪▪^])	BM32 vaccine induced allergen-specific IgG, improved clinical symptoms of seasonal grass pollen allergy and was well tolerated Two years AIT with BM32 induced continuously increasing allergen-specific IgG4, lacked IgE reactivity and induced only minor allergen-specific T-cell and cytokine responses AIT with BM32 established allergen tolerance in relation to conventional allergen extract AIT. It induced IL-10-secreting T cells and it reduced IL-5 Th2 –secreting cells BM32 vaccine induced hepatitis B-specific immune responses protecting against hepatitis B virus infection BM32 vaccine induced preS-specific IgG1 and IgG4 against the receptor binding for all eight HBV genotypes. The strongest levels of IgG antibodies were induced after five monthly BM32 injections
Synthetic hypoallergenic peptides	Route: ID Description: DBPC with synthetic peptide T-cell epitopes (Cat-PAD) from the major cat allergen Fel d 1. Mechanistic study with 12 Cat-PAD-treated subjects and 13 placebo-treated participants Reference: Rudulier CD *et al.*[70^▪^]	Peptides from Fel d 1 had no significant difference in the frequency of Fel d 1 CD4+ cells but may decrease the expression of CRth2 on the cell surface
	Route: SCIT Description: Exploratory study of a DBPC randomized phase IIb study with Bet v 1 contiguous overlapping peptide immunotherapy Study: 240 patients randomized for: Bet v 1 COPs 50 μg [78], Bet v 1 COPs 100 μg [81] or placebo [78] treatment Primary outcome: combined rhinoconjunctivitis symptom score and rhinoconjunctivitis MS during birch pollen season Reference: Kettner *et al.*[71^▪^]	Study participants previously treated with Bet v 1 COPs reported improved RSMS after the second birch pollen season following treatment Increase of Bet v 1-specific IgG4 antibodies and no long-term changes in Bet v 1 specific IgE antibodies
AIT by passive immunization	Route: SCIT Description: phase 1b DBPC with REGN1908–1909, Fel d 1-specific IgG Study: 73 patients: 600 mg REGN1908–1909 (*n* = 36) and placebo (*n* = 37) Primary outcome: change in total nasal symptom score (TNSS) Reference: Orengo *et al.*[77]	Significant reduction in TNSS after a single dose of REGN1908–1909 Sustained reduction in wheal diameter after treatment REGN1908–1909
Nucleic acid vaccines for AIT	Route: IM Description: phase I study assessing safety and immunological effects of the investigational CryJ2-LAMP DNA vaccine for Japanese Red Cedar (JRC) pollen-induced allergy Study: 24 patients, JRC non atopic (*n* = 6), JRC and/or Mountain Cedar (MC) into two separate cohorts of *n* = 9 per cohort Primary outcome: evaluation of safety and functional biomarkers Reference: Su *et al.*[85]	All subjects tolerated CryJ2-LAMP vaccinations well, indicating safety Majority of JRC sensitive and MC-sensitive subjects experienced skin test negative conversion Majority of treatment-emergent adverse events (TEAEs) reported were mild and most were injection site erythema
Virus-like nanoparticles	Route: IN Description: prophylactic vaccination of humanized mouse model with VNP-exposed or shielded VNP Art v 1. Experimental study of virus-like nanoparticles for allergy vaccines Reference: Kratzer B *et al.*[89^▪^]	VNP with shielded version of Art v 1 were hypoallergenic as shown by reduced degranulation of rat basophil leukemia cells sensitized with Art v 1-specific mouse VNP with shielded version of Art v 1 increased Foxp3+ Treg in lungs and cytokines were shifted towards Th1/Treg profile Surface exposed VNP-induced allergen-specific antibodies in mice
	Route: IM Description: evaluation of a prophylactic virus-like particle vaccine against a major house dust mite allergen, Der p 2, in a murine model Reference: Soongrung *et al.*[90^▪^]	Vaccinated mice later challenged with Der p 2 had a significantly lower immune response as measured by allergen- specific IgG1 and IgE antibodies compared with allergic control mice
Cell-based therapy	Route: bone marrow transplant Description: proof of concept studying evaluating potential of cell-based therapy in allergy prevention Treatment of mice with Phl p 5 bone marrow to suppress allergic immune response Reference: Baranyi *et al.*[91]	Mice, which received Phl p 5 bone transplantation did not develop Phl p 5-specific IgE upon multiple sensitizations T-cell responses and airway inflammation to Phl p 5 was also prevented
Prophylactic AIT vaccination	Route: SCIT Description: DBPC clinical study with nonallergic subjects who were vaccinated with recombinant hypoallergenic derivatives of the major birch pollen allergen, Bet v 1 for 2 years Study: 16 patients, 6 actively treated patients and 10 placebo-treated patients Primary endpoint: development of Bet v 1-specific IgG antibodies Reference: Campana *et al.*[96^▪▪^]	Induction of Bet v 1-specific IgG antibodies in nonallergic vaccinated subjects These specific-IgG antibodies were able to inhibit binding of IgE from birch allergic patients to Bet v 1

AIT, allergen-specific immunotherapy; DBPC, double-blind, placebo-controlled; ILIT, intralymphatic immunotherapy; SCIT, subciutaneous AIT; SLIT, sublingual immunotherapy.

**Box 1:**
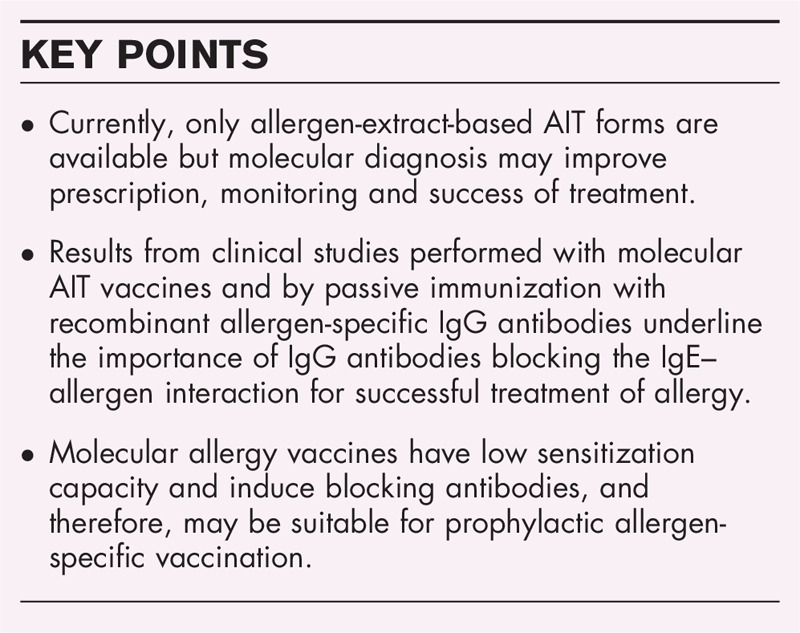
no caption available

## MOLECULAR ALLERGY DIAGNOSIS FOR PRESCRIPTION, MONITORING AND FOR ENHANCING TREATMENT SUCCESS OF **ALLERGEN-SPECIFIC IMMUNOTHERAPY**

On the basis of the fact that allergen sources contain allergen molecules, which are source-specific but also cross-reactive with unrelated sources, it has been proposed to use them for molecular diagnosis to better identify the genuinely sensitizing allergen sources for refined prescription of the AIT vaccines [18,19]. The advantage of defining the originally sensitizing allergen sources avoids that unnecessary administration of incorrect allergy vaccines is performed reducing possible side effects as well as treatment costs [20]. Initially this concept was misunderstood as it was thought that correct prescription would also increase efficacy of AIT but efficacy depends also on the quality of the AIT vaccine and the immune response of the treated patient to the vaccine. It was, therefore, also recommended to monitor the development of protective allergen-specific IgG antibodies in the course of the treatment, which by now is an accepted surrogate marker for the effects of AIT [8^▪▪^,^21^]. The advantage of molecular testing for AIT-induced IgG is that it allows identifying if a patient develops protective IgG against those allergens against which the patient is sensitized [22^▪▪^,^23^^▪▪^]. Using allergen microarrays containing small amounts of immobilized allergens, it was found that the development of blocking IgG antibodies is reflected by a decrease of allergen-specific IgE binding through competition of IgG with IgE for the binding sites on the allergens. This decrease of the IgE binding also serves as surrogate marker for effective AIT [24–26].

In the meantime, molecular allergy diagnosis has been implemented as companion diagnostic tool for AIT [27^▪^]. Multiallergen testing with allergen micro-arrays allows discriminating polysensitized from oligosensitized and monosensitized patients of whom the first may benefit more from symptomatic treatment whereas the latter may be more suitable for AIT (Fig. 1).

Several reports show that clinicians can use molecular testing for refining AIT prescription and there is evidence that it reduces treatment costs [28–30] (Fig. 1). Very recently two studies have pointed out additional advantages of molecular diagnosis for AIT prescription. These studies demonstrated that patients who were sensitized to allergens, which were included in allergen extract-based vaccines had a better treatment success than patients who were sensitized also to other allergen molecules not included in the vaccines [22^▪▪^,^23^^▪▪^] suggesting that clinical outcomes might be improved by selecting AIT extracts fitting the patient's sensitization profile [22^▪▪^,^23^^▪▪^]. Another interesting diagnostic option are chips-containing micro-arrayed peptides derived from human rhinovirus-strains, which together with allergen chips can be used for the differential diagnosis of allergen-induced and rhinovirus-induced asthma [4^▪^,11^▪^,^31^,^32^].

## ALLERGEN EXTRACT-BASED **ALLERGEN-SPECIFIC IMMUNOTHERAPY** VACCINES ADMINISTERED BY ALTERNATIVE ROUTES

Although everybody is aware of the limitations of allergen extracts [10], several approaches are ongoing regarding AIT with allergen extracts that are administered via alternative routes (Fig. 2 and Table 1). A recently published clinical phase III study performed as international, double-blind, placebo-controlled (DBPC) clinical trial investigated the clinical effects by assessing the total combined score in more than 1400 house dust mite allergic patients (Table 1). Patients were randomized into a placebo and a SLIT group receiving a tablet containing a mixture of *Dermatophagoides pteronyssinus* (Dp) and *Dermatophagoides farinae* (Df) extract for 1 year. Active treatment achieved a relative least squares mean difference of 16.9% improvement of the total combined score in the tablet versus placebo group [33^▪▪^]. The latter study reflects well the magnitude of effect of SLIT with tablets observed in earlier large AIT trials [34]. Like in other studies, there was a very low increase of allergen-specific IgG levels in the active group but a strong increase of allergen-specific IgE levels from median Dp-specific baseline levels of 14.81 kUA/l to median levels of Dp-specific IgE of 44.01 kUA/l was found. Treatment-related adverse events, mainly application site reactions occurred in more than 50% of the actively treated patients but were rare in the placebo group raising the question if SLIT studies can be effectively blinded.

Two recent intralymphatic immunotherapy (ILIT) studies showed that ILIT is clinically effective but the treatment-induced IgG antibody levels were not higher than in subcutaneous AIT (SCIT) studies and in one study no effective boost of antibody levels by the booster injection was observed [35^▪▪^,^36^^▪▪^] (Table 1). Therefore, it remains unclear whether ILIT has any advantages over SCIT.

Data from epicutaneous AIT (EPIT) studies performed with peanut allergen extracts provide evidence for clinical effects [37^▪▪^,^38^^▪▪^] (Table 1) but these effects are modest, and a systematic review and metaanalysis [39] concludes that more studies are necessary to understand if this form of AIT is effective. Furthermore, the mechanisms of EPIT are not clear as it has been demonstrated that epicutaneous allergen administration induces no relevant increases of allergen-specific IgG antibodies but rather increases allergen-specific T-cell activation [40]. Viaskin is an epicutaneous immunotherapy formulation [41] currently in three different phase III clinical trials for pediatric patients and it is yet to receive approval for use and marketing. It has so far demonstrated some low induction of IgG_4_ antibodies to certain peanut allergens [42^▪▪^]. However, regulatory authorities have questioned its efficacy [43].

Oral immunotherapy (OIT) is mainly conducted with digestion-resistant food allergens [44] but does not seem to be applicable for respiratory allergy. Food OIT studies indicate that beneficial effects depend on the induction of allergen-specific IgG antibodies [45^▪▪^] (Table 1). Palforzia from Aimmune Therapeutics (Brisbane, CA, USA) and an OIT formulation consisting of peanut protein increased in 67.2% the amount of peanut that patients could consume in a clinical trial [46]. However, it is still not without the risk of causing anaphylaxis and patients must still avoid a peanut diet but it is approved for use and marketing. However, a major disadvantage of OIT is that it induces severe side effects [47].

## ’MOLECULAR APPROACHES’ WITH NATURAL ALLERGEN PREPARATIONS

Several early studies have shown that AIT with purified major allergens or chemically modified major allergen preparations is clinically effective [48–50]. In this context, the AIT study performed by Pauli *et al.*[51], should be mentioned, which showed that AIT with natural purified major birch pollen allergen, Bet v 1 and recombinant Bet v 1 was as effective as AIT with birch pollen allergen extract. A recent DBPC AIT trial performed with the purified major allergen of *Alternaria alternata*, Alt a 1, demonstrated reductions in the allergic symptoms scores, as well as IgE levels, and increased IgG_4_ antibodies in allergic patients [52^▪▪^] (Fig. 2 and Table 1).

Another allergen extract-based approach has utilized a hydrolyzed allergen extract from *Lolium perenne* including peptides of a size between 1 and 10 kDa, which were administered without adjuvant. Initial studies indicated that the vaccine induces allergen-specific IgG responses [53^▪▪^] and reduces allergic inflammation [54,55] (Table 1).

Despite the use of allergen peptides, which were thought to be hypoallergenic severe side effects were observed in the course of a randomized, multicenter DBPC trial [55]. There was evidence for clinical efficacy in a subset of patients but the phase III trial results did not meet the endpoints (https://www.businesswire.com/news/home/20191124005103/en/ASIT-biotech-gp-ASIT%E2%84%A2-Phase-III-Trial-Grass).

## RECOMBINANT HYPOALLERGENIC MOLECULES

The first generation of recombinant hypoallergenic molecules has been made to reduce IgE reactivity and allergenic activity (i.e. the ability of a molecule to induce IgE-dependent mast cells/basophil activation) of the molecules and at the same time to maintain allergen-specific T-cell epitopes [56] (Fig. 2). The first AIT trial, which was conducted with hypoallergenic derivatives of the major birch pollen allergen Bet v 1 indeed demonstrated that this approach may be effective and avoids immediate type side effects [57] but late phase side effects, were still observed [58]. Atopy patch test studies indicated that these late phase side effects are because of T-cell activation [59,60]. Accordingly, a refined second generation of recombinant hypoallergens was engineered to reduce the presence of allergen-specific T-cell epitopes but to maintain the ability of the derivatives to induce allergen-specific IgG antibodies [61] (Fig. 2). For this purpose, fusion proteins consisting of a nonallergenic carrier protein and nonallergenic peptides derived from the IgE epitopes of allergens were produced. A virus-derived carrier molecule provided additional T-cell help for the production of allergen-specific IgG production without activating allergen-specific T cells [62]. For the peptide-carrier fusion proteins made for treatment of grass pollen allergy (i.e. the BM32 vaccine) using the hepatitis B (HBV)-derived PreS protein as carrier it could be shown, that the PreS-containing vaccine induced also HBV-specific antibodies, which protected against HBV-infection *in vitro*[63]. In the meantime, the BM32 vaccine has been shown to be clinically effective in a multicenter DBPC phase IIb study [64] (Table 1) and is scheduled for phase III studies. The analysis of sera from a recent phase IIb study confirmed the robust induction of IgG antibodies against the domain of PreS, which is critical for the binding of the HBV virus to liver cells and demonstrated cross-reactivity with the most common HBV strains [65^▪▪^] (Table 1). Thus PreS-based allergy vaccines may be useful for vaccination against HBV.

Some additional results for BM32 were obtained in sub-studies. One sub-study compared the magnitude and specificity of allergen-specific IgG responses in patients treated with BM32 and a traditional, allergen extract-based registered SCIT [66^▪▪^] (Table 1). It was found that much less injections of BM32 compared with the extract-based SCIT were needed to induce a comparable IgG response and the BM32 vaccination-focused IgG antibodies precisely to the IgE binding sites of the allergens. Another sub-study demonstrated that vaccination with BM32 induced continuously growing allergen-specific IgG_4_ responses and did not activate allergen-specific T-cell responses [67^▪▪^] (Table 1).

A few preclinical results describing recombinant vaccine constructs may also be mentioned. For example, basophil activation experiments demonstrated that a hybrid consisting of major allergens from two different aeroallergen sources, Bet v 1 and Phl p 5 induced a much reduced activation, compared with the monomeric forms of the proteins [68]. Similarly, fusion proteins consisting of four antigenic regions from the major allergens of the mite species *D. pteronyssinus* showed a significantly reduced IgE reactivity, compared with the individual proteins (Der p 1, Der p 2 and Der p 7) [69]. Mice immunized with the HDM hybrid protein generated blocking IgG antibodies, thus showing a possibility for a novel HDM vaccine [69].

## SYNTHETIC HYPOALLERGENIC PEPTIDES

Allergen-derived synthetic peptides containing T-cell epitopes and lacking IgE reactivity have been considered for AIT for almost 30 years. This approach was thought to induce T-cell tolerance with the hope that it would reduce allergen-specific IgE production and induce protective allergen-specific IgG responses. The AIT studies performed in experimental animal models and in clinical trials in humans provide indeed evidence that peptide-based AIT may induce signs of immunological tolerance (Fig. 2 and Table 1). In a recent study, a downregulation of a chemokine receptor, chemoattractant receptor-homologous molecule expressed on Th2 cells (CRTh2) in patients that had received a Fel d 1 peptide vaccine (Cat-PAD) was reported [70^▪^]. This mechanistic sub-study was part of a phase III trial, which showed no effects of treatment over placebo [70^▪^]. The AIT trials performed with T-cell epitope-containing peptides could never show unambiguous effects of treatment on the production of allergen-specific IgE or IgG whenever performed with short peptides.

An induction of allergen-specific IgG antibodies was only observed with longer, immunogenic peptides as reported in a phase IIb trial performed with contiguous overlapping peptides from Bet v 1 [71^▪^] (Fig. 2 and Table 1). In one of the studies, a significant clinical improvement of more than 20% compared with the placebo group was observed. This was associated with increased Bet v 1-specific IgG_4_ antibodies up to two pollen seasons after treatment [71^▪^] (Table 1). It thus seems that AIT with allergen-derived peptides requires the induction of allergen-specific IgG to achieve clinical benefit and that such an effect can only be obtained with long and immunogenic peptides.

Although the induction of T-cell tolerance by peptide AIT remains challenging, one may consider this approach for preventive tolerance induction. In this context, approaches, such as prophylactic oral tolerance induction with T-cell epitope-containing peptides have been reconsidered for allergen-specific prevention [72]. A recent study performed in murine model of fish allergy showed that prophylactic feeding with the major fish allergen parvalbumin induced tolerance point to a T-cell-mediated, tolerogenic effect [73^▪^]. However, further studies will be necessary to investigate if such an effect can also be obtained with synthetic allergen-derived peptides. Another recent study prepares the ground for such experiments by defining a panel of synthetic hypoallergenic peptides covering the major house dust mite allergens, Der p 1, Der p 2, Der p 5, Der p 7, Der p 21 and Der p 23 [74].

## IMMUNOTHERAPY BY PASSIVE IMMUNIZATION

In 1935, it was shown by Cooke *et al.*[75] that serum from AIT-treated patients containing allergen-specific IgG protects against allergic skin inflammation. This study confirmed the earlier work by Dunbar in 1903 [76], which demonstrated that pollen allergen-specific antisera neutralized the allergenic activity of pollen allergens. These historic studies indicated that it may be possible to use allergen-specific IgG antibodies, which block allergic patients IgE binding to allergens for treatment of allergy by passive immunization.

The great potential of the passive immunization approach was recently shown in a clinical study performed in cat allergic patients who were passively vaccinated with monoclonal IgG antibodies against the major cat allergen, Fel d 1 [77] (Fig. 2 and Table 1). The injection of antibodies improved the allergic symptoms of the allergic patients, caused few side effects and a correlation between clinical symptoms and the IgG/IgE ratio was shown. The passive immunization approach is also supported by experimental animal studies.

It was shown in a murine model of fish allergy that passive immunization with IgG antibodies specific for a hypoallergenic mutant of the major fish allergen, parvalbumin reduced allergic symptoms [78]. In another study performed in a model of peanut allergy, transfer of allergen-specific IgG antibodies led to a decreased response in local and systemic reactions after allergen challenge with peanut extract [79^▪^].

Major challenges for such approach are the high costs required for the production of large quantities of antibodies and the fact, that for certain allergen sources containing more than one major allergen, cocktails of several monoclonal antibodies will be needed to achieve sufficient blocking of IgE binding to each of the allergens. Moreover, certain allergens contain multiple IgE epitopes which could not be neutralized even with several different monoclonal antibodies [80].

## NUCLEIC ACID VACCINES FOR **ALLERGEN-SPECIFIC IMMUNOTHERAPY**

Nucleic acid vaccines are based on the application of DNA or RNA to produce the antigen in transfected cells of the host instead of immunizing with the antigen. This approach was already described in 1992 [81]. In 1993, it was demonstrated that the injection with DNA encoding a viral protein induced protection by boosting both T cell and antibody responses [82]. The approach was then tested for allergy in animal models and it was shown that it could mitigate an allergen-specific immune response [83,84] (Fig. 2). However, the technology was tested only recently in clinical trials. A phase 1A and 1B study was performed with the Japanese cedar pollen DNA vaccine encoding the major allergen Cry j 2. The study was conducted in a region where no Japanese cedar trees were growing, therefore, natural exposure could not be determined. Additionally, the study did not include a mock control group and the treated patients experienced 88 different adverse events in total [85] (Table 1).

A drawback of genetic vaccination is the weak induction of blocking IgG antibodies. Therefore, several methods regarding the route of delivery have been studied, such as gene gun vaccination or electroporation [86].

Other possible problems with nucleic acid-based vaccination may result from the integration of the vaccine into the genome, the long-term effects of the plasmid DNA, the development of anti-DNA antibodies and the long-term expression of the allergen encoded, which could lead to the development of adverse events or even more severe inflammatory diseases [87].

## VIRUS-LIKE NANOPARTICLES

Using a model of mugwort pollen allergy [88], it was demonstrated that inclusion of the complete major mugwort pollen allergen, Art v 1 within VNPs resulted in a nonanaphylactogenic vaccine when tested in basophil activation tests [89^▪^] (Fig. 2 and Table 1). Testing of the vaccine in a preclinical model of mugwort pollen [89^▪^] and house dust mite allergies showed that the VNPs may be used for prophylactic vaccination [89^▪^,90^▪^] (Table 1). In a preclinical study with a vaccine candidate against peanut allergy where Ara h 1 or Ara h 2 were coated to the cucumber mosaic virus-derived particle (CMV), positive results were obtained. The vaccine-induced protective responses in mice [79^▪^] (Table 1). However, the VNP approach has so far been only tested in preclinical models, the vaccines may be difficult to produce under Good Manufacturing Practice (GMP) conditions suitable for clinical trials, and accordingly there is so far no experience in clinical AIT studies.

## CELL-BASED THERAPY

Different cell-based approaches for prevention and treatment of allergy are emerging (Fig. 2 and Table 1). One approach is based on the prophylactic administration of allergen-expressing leukocytes into new-born recipients with the goal to induce allergen-specific immune tolerance at the cellular and humoral levels. Results from experimental animal studies provide evidence that such a treatment can prevent the development of allergic sensitization [91]. Another different approach utilizes chimeric antigen receptor (CAR) T cells for targeting cells of the allergic immune response. CAR T cells were originally made for cancer immunotherapy and are engineered to express an immunoreceptor, which can specifically recognize certain targets on cells and are linked with T-cell activating functions. Several applications for the treatment of allergy and asthma by CAR T cells have been discussed [92^▪^]. For example, it has been suggested to engineer CAR T cells to target cells expressing membrane-bound IgE (mIgE) similar as has been done with a monoclonal antibody-based therapy earlier [93]. Although the CAR T-cell approach is currently considered to be used as a nonallergen-specific form of treatment, it may be envisaged that this approach could be used to target also allergen-specific T cells or B cells producing allergen-specific IgE via the variable regions of the T-cell receptors or membrane IgE but the polyclonality of allergen-specific T-cell receptors will be a major hurdle for an allergen-specific approach. In addition, CAR T-cell approaches may cause side effects, such as inflammation, neurotoxicity, anaphylaxis, immunological rejection, cellular injury, insertional oncogenesis and cytokine storm (CRS) [92^▪^].

## PROPHYLACTIC ALLERGEN-SPECIFIC VACCINATION

There is growing evidence that prophylactic allergen-specific vaccination may be a possibility for preventing allergies. Data obtained by studying allergic sensitization at the molecular level in birth cohorts suggest several windows of opportunity for interrupting or preventing the process of allergic sensitization by tolerance induction or allergen-specific vaccination [94] (Table 1). A recent perspective article has highlighted the possible steps for developing and evaluating prophylactic vaccination concepts and suggests that recombinant hypoallergenic molecules may be best suited for preventive vaccination [95^▪^].

In this context, a first controlled vaccination study performed with hypoallergenic recombinant allergen derivatives of the major birch pollen allergen, Bet v 1 in nonallergic individuals should be mentioned (Table 1). This study [96^▪▪^] demonstrated that vaccination with the hypoallergenic Bet v 1 derivatives could induce normal Bet v 1-specific IgG responses in nonallergic individuals and that these IgG antibodies blocked allergic patients’ IgE recognition of Bet v 1. Even more important, this study showed that vaccination with the hypoallergenic derivatives did not induce allergic sensitization in the nonallergic individuals [96^▪▪^]. The study may thus be considered as a first step towards prophylactic allergen-specific vaccination.

## CONCLUSION

AIT is an effective, cost-effective and disease-modifying treatment for allergy with long-lasting effects. It is ideally suited for a precision medicine approach for managing allergic diseases. Molecular allergy diagnosis is useful for prescription, monitoring and even for prediction of treatment success and has become an integral part of modern allergy diagnosis. Modern forms of molecular vaccines are urgently needed to improve AIT, to render it useful for broad application to respiratory, food and other forms of allergy and for prophylactic use. Certain molecular AIT concepts have been successful in clinical trials and need to be carried forward vigorously to catch up with advances made in molecular allergy diagnosis to provide best practice precision medicine for allergic patients.

## Acknowledgements

None.

### Financial support and sponsorship

This work was supported by the MCCA PhD-program, grant F4605 from the Austrian Science Fund (FWF) and by the Country of Lower Austria.

### Conflicts of interest

R.V. has received research grants from the Austrian Science Fund (FWF), from Biomay AG, Vienna, Austria and from Viravaxx, Vienna, Austria. He serves as a consultant for Viravaxx, Vienna, Austria. The other authors have no conflict of interest to declare.

## REFERENCES AND RECOMMENDED READING

Papers of particular interest, published within the annual period of review, have been highlighted as:

▪ of special interest
▪▪ of outstanding interest
